# Provision of palliative and end-of-life care in UK care homes during the COVID-19 pandemic: A mixed methods observational study with implications for policy

**DOI:** 10.3389/fpubh.2023.1058736

**Published:** 2023-03-14

**Authors:** Andy Bradshaw, Sophia Ostler, Claire Goodman, Izabele Batkovskyte, Clare Ellis-Smith, India Tunnard, Anna E. Bone, Stephen Barclay, Martin Vernon, Irene J. Higginson, Catherine J. Evans, Katherine E. Sleeman

**Affiliations:** ^1^King's College London, Cicely Saunders Institute of Palliative Care, Policy and Rehabilitation, London, United Kingdom; ^2^Center for Research in Public Health and Community Care (CRIPACC), University of Hertfordshire, Hatfield, United Kingdom; ^3^Primary Care Unit, Department of Public Health and Primary Care, University of Cambridge, Cambridge, United Kingdom; ^4^Tameside and Glossop Integrated Care NHS Foundation Trust, Ashton-under-Lyne, United Kingdom; ^5^Sussex Community NHS Foundation Trust, Brighton, United Kingdom

**Keywords:** care homes, palliative care, end-of-life care, COVID-19, policy, mixed methods, observational

## Abstract

**Introduction:**

Little consideration has been given to how the provision of palliative and end-of-life care in care homes was affected by COVID-19. The aims of this study were to: (i) investigate the response of UK care homes in meeting the rapidly increasing need for palliative and end-of-life care during the COVID-19 pandemic and (ii) propose policy recommendations for strengthening the provision of palliative and end-of-life care within care homes.

**Materials and methods:**

A mixed methods observational study was conducted, which incorporated (i) an online cross-sectional survey of UK care homes and (ii) qualitative interviews with care home practitioners. Participants for the survey were recruited between April and September 2021. Survey participants indicating availability to participate in an interview were recruited using a purposive sampling approach between June and October 2021. Data were integrated through analytic triangulation in which we sought areas of convergence, divergence, and complementarity.

**Results:**

There were 107 responses to the survey and 27 interviews. *We found that* (i) relationship-centered care is crucial to high-quality palliative and end-of-life care within care homes, but this was disrupted during the pandemic. (ii) Care homes' ability to maintain high-quality relationship-centered care required key “pillars” being in place: integration with external healthcare systems, digital inclusion, and a supported workforce. Inequities within the care home sector meant that in some services these pillars were compromised, and relationship-centered care suffered. (iii) The provision of relationship-centered care was undermined by care home staff feeling that their efforts and expertise in delivering palliative and end-of-life care often went unrecognized/undervalued.

**Conclusion:**

Relationship-centered care is a key component of high-quality palliative and end-of-life care in care homes, but this was disrupted during the COVID-19 pandemic. We identify key policy priorities to equip care homes with the resources, capacity, and expertise needed to deliver palliative and end-of-life care: (i) integration within health and social care systems, (ii) digital inclusivity, (iii) workforce development, (iv) support for care home managers, and (v) addressing (dis)parities of esteem. These policy recommendations inform, extend, and align with policies and initiatives within the UK and internationally.

## Introduction

Internationally, the burden of COVID-19 deaths fell disproportionately on care homes. The World Health Organization estimated that between March 2020 and February 2021, care home residents made up 41% of all pandemic-related deaths worldwide (1). In the UK, there was a 220% rise in care home deaths during the first pandemic wave, temporarily making them the most common place to die (2), a situation that had not been projected to be reached until 2040 (3).

Within an international context, the term “care home” generally refers to long-term care provision for adults who require 24-h assistance with personal care and daily living activities. The majority of residents are older people (typically over 80) in the last 1 or 2 years of life who live with multiple long-term progressive health conditions, often including dementia (4). Because of this, palliative and end-of-life care is a central element of care provision in care homes (5). This approach to care aims to improve quality of life through the adoption of holistic, person-centered, and multidisciplinary care processes for people with complex long-term, life-limiting, or acute life-threatening conditions (6).

From the start of the COVID-19 pandemic, the high number of deaths occurring in care homes was the subject of intense policy, media, and research scrutiny. This focused on infection prevention/control, including testing for COVID-19, visiting restrictions, and personal protective equipment, as well as the psychological impact of the pandemic on staff, residents, and family carers (7–13). However, research examining the impact of the COVID-19 pandemic on the provision of palliative and end-of-life care in care homes has been limited (14). Research in this area has focused on specific disease types (such as dementia) (15, 16), or on specific elements of palliative care such as anticipatory prescribing (17), early bereavement, (18) advance care planning (19), and the response of care home managers (20). Some of these studies have highlighted how, during the pandemic, care home residents were more likely to have poorer experiences of palliative and end-of-life care compared to other care settings (18, 21).

Developing a more comprehensive understanding of how care homes within the UK responded to and experienced the rapid rise in the need for palliative and end-of-life care during the pandemic is important. This is so that we can identify opportunities for strengthening the provision of this type of care in care homes to meet future demographic challenges. The aims of this study were to: (i) investigate the response of UK care homes in meeting the rapidly increasing need for palliative and end-of-life care for residents during the COVID-19 pandemic and (ii) propose recommendations for strengthening the provision of palliative and end-of-life care within care homes.

## Methods

### Study design and participant recruitment

This paper presents the results from the CovPall Care Homes study, which was a mixed methods observational study consisting of two work packages: (i) an online cross-sectional survey of UK care home practitioners and (ii) in-depth qualitative interviews with care home practitioners. The rationale for using a mixed methods design was to integrate and triangulate both qualitative and quantitative methods and to explore the research aims in a depth and detail that would not be possible using one approach alone (22).

**Figure 1:**
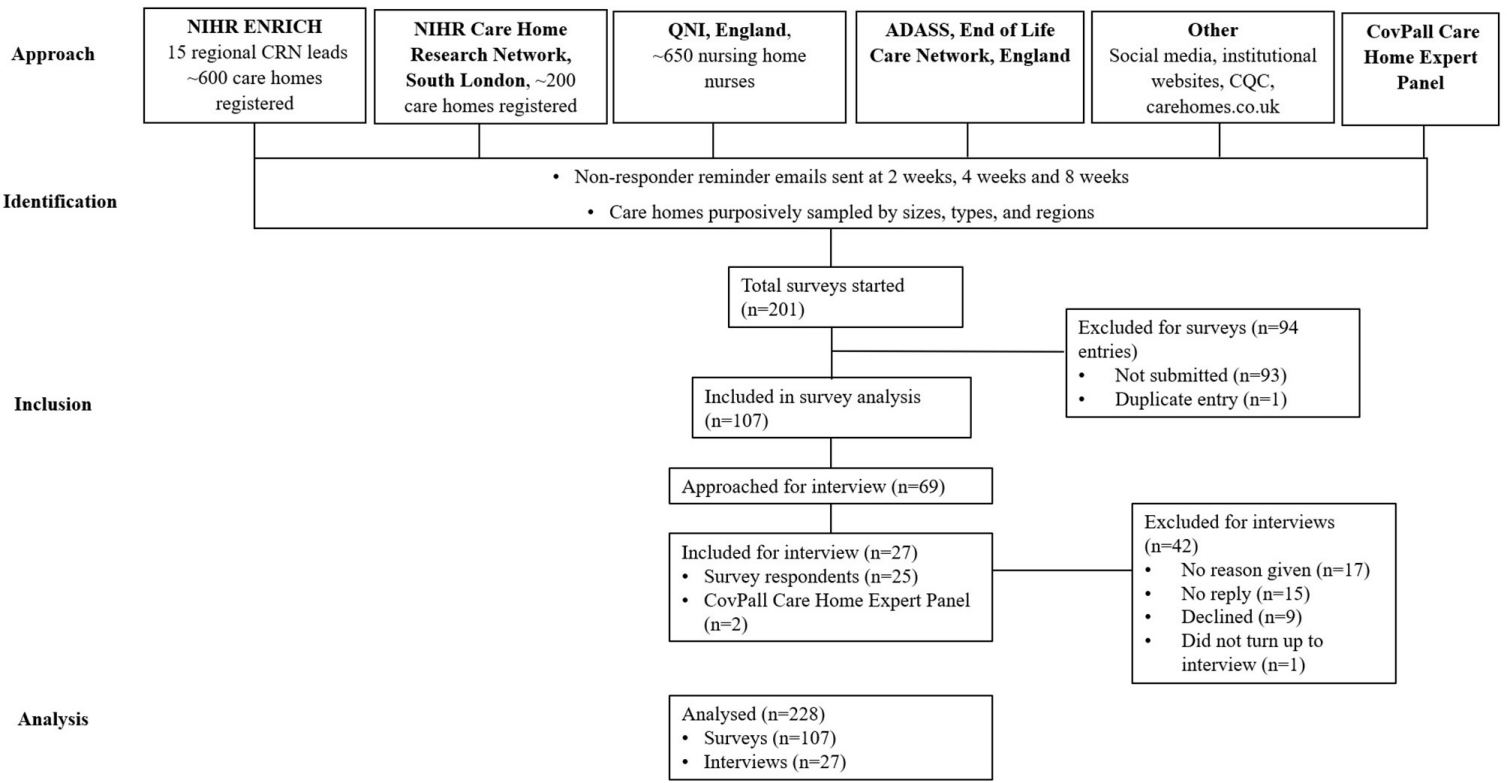
Recruitment flow diagram.

We used purposive sampling to ensure representation of different sizes, types, and regions from a sampling frame of all UK care homes with or without on-site nursing. Care homes were identified and recruited via our institutional websites, social media, and through working with established national care home practitioner networks. Care home managers or their nominees were invited to complete an online survey, with up to three reminder emails sent (see Figure 1 for recruitment flowchart). Recruitment and distribution of surveys took place between April and September 2021. By completing and submitting the survey, participants provided consent to participate in this study. The survey reporting was in line with the STROBE checklist (23).

For qualitative interviews, care home practitioners who had completed the survey and had agreed to be contacted were purposively sampled (using the same criteria). Additional interviewees were recruited through the study Care Home Expert Panel. Informed consent was confirmed virtually prior to the interview by the researcher (IT, IB, or LB). The researcher talked through each item on the consent form with the interview participant, confirmed verbally that they agreed (or not), marked the responses on the consent form, and recorded the responses in a separate digital file from the interview. A copy of the consent form was emailed to the participant. Participant recruitment and interviews ran concurrently between June and October 2021. Recruitment continued until all networks, avenues, and potential recruitment opportunities had been exhausted.

### Data collection

The survey design was informed by a rapid review of evidence on the provision of care in care homes during the pandemic. We tested and refined the survey with our Care Home Expert Panel, Patient and Public Involvement group (most of whom were family carers with experience of care homes), and Study Steering Committee (comprising care home experts, policy makers, and informal carers). The survey included open- and closed-ended questions that sought information on the respondent role, type of care home, and the impact of COVID-19 on the provision of palliative and end-of-life care (see Supplementary material 1 for the full survey). REDCap was used to securely build and host the survey, which was disseminated and filled in online. Invitations to complete the survey were disseminated via the networks and collaborators outlined in Figure 1 between April and September 2021. Missing/incomplete data were followed up by contacting participants where necessary.

For the second work package, individual semi-structured qualitative interviews were conducted online using Zoom. The interview guide (see Supplementary material 2) was developed using preliminary data from the survey to allow in-depth exploration of the experiences of practitioners in providing palliative care in care homes during the first two waves of the pandemic. The interviews were conducted by three researchers (IT, IB, and LB) and were digitally recorded, anonymized, and transcribed verbatim. On average, they lasted a median of 46 min (range = 22–83 min).

### Data analysis

Initially, the different forms of data were analyzed individually. Numerical survey data were analyzed descriptively in SPSS (v27). Qualitative interview transcripts and free-text survey data were analyzed inductively (by SO and AB) using reflexive thematic analysis grounded in a constructionist paradigm (24). The analysis comprised six iterative steps in which SO and AB: (i) familiarized themselves with the data; (ii) generated initial codes by labeling segements of transcripts that aligned with our research aims; (iii) generated intial themes by grouping codes into categories and categories into themes that told us something important about the study aims; (iv) reviewed and revised themes through iterative group discussions with authors and members of the Care Home Expert Panel and Public Study Reference Panel; (v) defined and named themes; and (vi) interpreted data and produced a findings report.

In combining data, we adopted a multiple perspectives approach (25). This entailed integrating and interpreting data through analytic triangulation, in which we sought areas of: (i) convergence, (ii) divergence, and (iii) complementarity. In this way, each form of data enriched the other, becoming more than the sum of their parts, and providing a richer understanding of the study aims (25). During the analytic process, we recognized that Donabedian's (26) model on structures and processes of care provided a useful lens to understand and interpret the findings. We used this model to ask further questions about the data, particularly on how COVID-19 impacted the structures and processes through which pallaitive and end-of-life care could be delivered by staff within care homes.

In contributing to a rigorous analysis, we used Braun and Clarke's assessment tool for quality reflexive thematic analysis to guide the analysis and write-up (27). A key step that we took throughout the analytic process was to draw on the wider research team, the Care Home Expert Panel, Patient and Public Involvement group, and Study Steering Committee as “critical friends” (28). This entailed working collaboratively by meeting regularly to discuss the ongoing data analysis, alongside giving written feedback over numerous iterations of the study findings. Through these processes, findings were constructively challenged, and alternative interpretations of the data were provided and integrated into the data analysis. These processes took place until the research team agreed that the analysis was an accurate reflection of the participants' accounts that addressed the study aims. This spirit of constructive collaboration was also used within a Policy Roundtable, which was convened in November 2021 to discuss the data and potential policy solutions.

The researchers involved in the data analysis also kept reflexive diaries in which they recorded initial “hunches” on what they felt were key messages within the data with regards to the study's aims (29). They also recorded their reflections both introspectively (inward reflections on how they impacted the research process and vice versa) and intersubjectively (reflections on relationships between them and participants) (30). The detail in the reflexive diaries was included in the data analysis as a “springboard for interpretations and more general insight” into the ways through which understandings of the research aim were being co-constructed through the research process (30).

### Ethical considerations

Institutional ethical approval was granted by the King's College London Research Ethics committee (LRS-19/20-18541). In this study, we also recognized ethics as a process and engaged in “ethics in practice” (31) as a way to navigate ongoing and potentially unexpected ethical issues that may have arisen throughout the research process. We appreciated that participants were working in an unprecedented context characterized by uncertainty, high pressure, and time limitations. To mitigate this, we worked with the Care Home Expert Panel group to ensure that the survey was concise, asked relevant questions, and used language that was accessible and sensitive.

During interviews, we recognized that there was a potential for participants to become distressed when reflecting on the challenges of the COVID-19 pandemic. There was also a potential for participants not wanting to disclose information that revealed risk or poor practice. To mitigate this, interview guides were developed and conducted in a sensitive and responsive manner, with clear messages that the content of conversations were confidential (unless they posed severe risks to their own or others' wellbeing) and participants' identities would remain anonymous. The research team also collated bereavement and support resources for practitioners that were shared with participants where appropriate.

We also appreciated the potential emotional impact that conducting interviews in this context may have had on researchers. Regular bi-weekly debriefing sessions were held between researchers (IT, IB, and LB) and a senior member of the research team (CE, who has experience in qualitative interviewing in sensitive contexts) in which issues, challenges, or problems that arose in interviews were discussed.

### Role of the funding source

The funder of the study, the National Institute for Health and Care Research Policy Research Programme, had no role in the study design, data collection, data analysis, data interpretation, or writing of the report.

## Results

**Figure 2:**
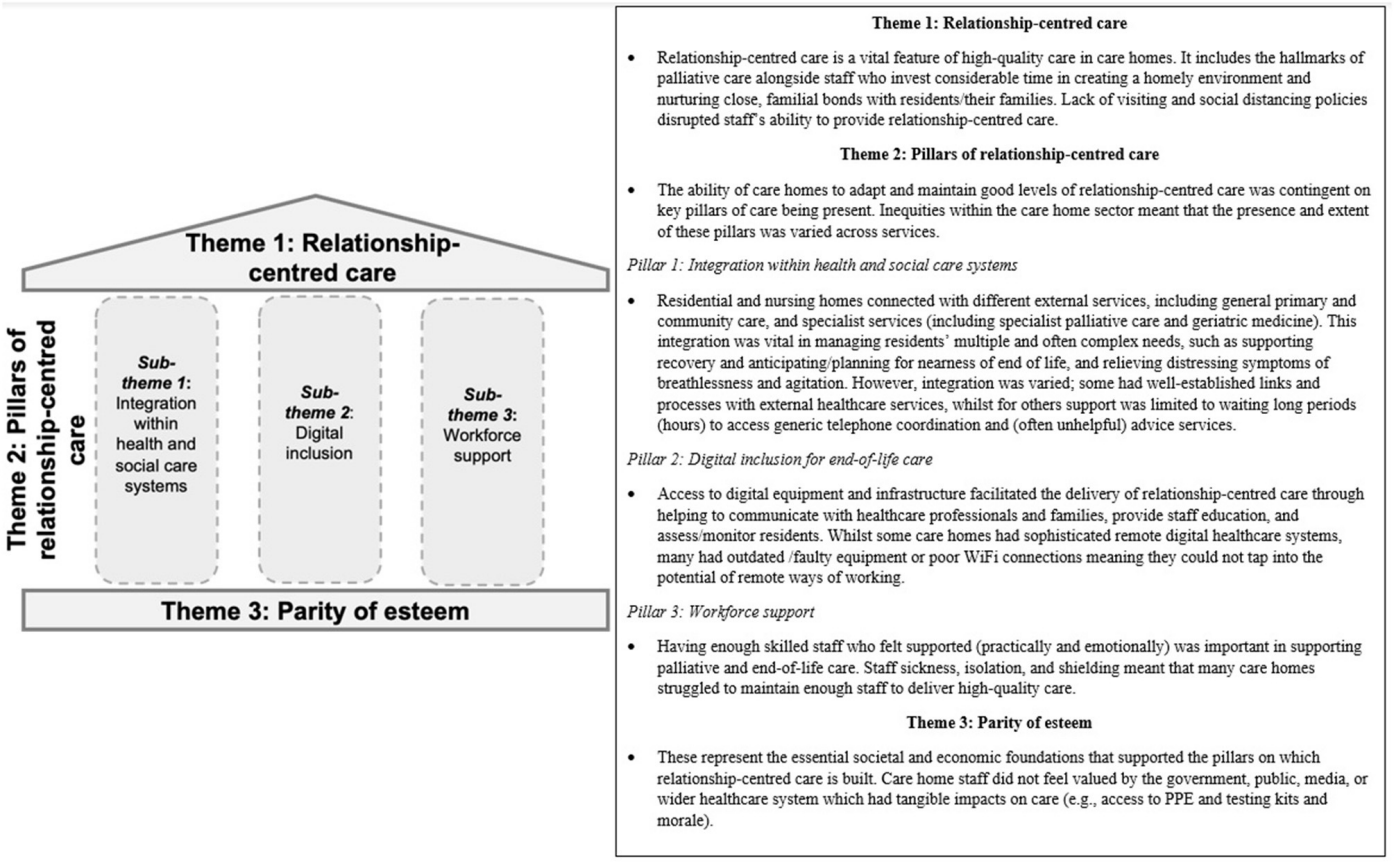
Overview of integrated findings.

A total of 107 participants completed the online survey, and 27 participated in qualitative interviews (out of 69 approached). Table 1 provides an overview of the participants. An overview of the descriptive survey findings can be seen in Table 2. Three themes were constructed following the triangulation of the survey and interview data (Figure 2).

**Table 1:** Survey respondent and interview participant details.

**Survey**	**Number**	**%**	**Interviews**	**Number**	**%**
**Total responses**			**Total responses**		
	107			27	
**Role**			**Role**		
Manager	77	72	Manager	16	59.2
Deputy manager	10	9.3	Deputy manager	3	11.1
Registered nurse	8	7.5	Registered nurse	4	14.9
Senior carer/team leader	2	0.2	Other	4	14.9
Other	11	10.3			
**Type of care home**			**Type of care home**		
Residential	49	45.7	Residential	6	22.2
Nursing	24	22.4	Nursing	10	37.0
Dual-registered for residential and nursing	34	31.7	Dual-registered for Residential and Nursing	11	40.7
**Size of care home**			**Region**		
Small (≤ 10 beds)	10	9.3	England	25	93.5
Medium (11–49 beds)	59	55.1	Devolved nations (Scotland, Wales, and Northern Ireland)	2	7.5
Large (50+ beds)	37	34.6	**Gender**		
Missing	1	0.9	Female	21	77.7
**Region**			Male	6	22.3
England	99	92.5			
Devolved nations (Scotland, Wales, and Northern Ireland)	8	7.5			

**Table 2:** An overview of descriptive survey findings.

	**Residential (49)**	**Nursing (24)**	**Both nursing and residential (34)**	**Total (107)**
**Confirmed or suspected outbreak of COVID-19? (** ***n*** **, %)**				
Yes	25 (51.0%)	19 (79.2%)	28 (82.4%)	72 (67.3%)
No	23 (46.9%)	5 (20.8%)	4 (11.8%)	32 (29.9%)
Missing	1 (2.0%)	0 (0.0%)	2 (5.9%)	3 (2.8%)
**Staff shortages**				
Yes	17 (34.7%)	13 (54.2%)	18 (52.9%)	48 (45.3%)
No	32 (65.3%)	11 (45.8%)	15 (44.1%)	58 (54.7%)
Missing	0 (0.0%)	0 (0.0%)	1 (2.9%)	1 (0.9%)
**During COVID-19 were there issues with:**				
Staff turnover	3 (6.1%)	3 (12.5%)	8 (23.5%)	14 (13.1%)
Staff redeployment	1 (2.0%)	3 (12.5%)	6 (17.6%)	10 (9.3%)
Staff absenteeism	15 (30.6%)	13 (54.2%)	18 (52.9%)	46 (43.0%)
Staff stress	31 (63.3%)	17 (70.8%)	27 (79.4%)	75 (70.1%)
Staff Suffering losses	6 (12.2%)	9 (37.5%)	14 (41.2%)	29 (27.1%)
**Did your care home allow visitors during the COVID-19 pandemic for people approaching the end of life?**				
Yes	37 (75.5%)	21 (87.5%)	26 (76%)	84 (78.5%)
No	11 (22.4%)	3 (12.5%)	7 (20.6%)	21 (19.6%)
Missing	1 (2.0%)	0 (0.0%)	1 (2.9%)	2 (1.9%)
**Did staff responsibilities in providing care for people nearing the end-of-life change?**				
Yes	15 (30.6%)	5 (20.8%)	16 (47.1%)	36 (33.7%)
No	33 (67.3%)	19 (79.2%)	16 (47.1%)	68 (63.6%)
Missing	1 (2.0%)	0 (0.0%)	2 (5.9%)	3 (2.8%)
**Changes in how often agency staff used:**				
Yes	20 (40.8%)	12 (50.0%)	17 (50.0%)	49 (45.8%)
No	28 (57.1%)	12 (50.0%)	15 (44.1%)	55 (51.4%)
Total (missing)	1 (2.0%)	0 (0.0%)	2 (5.9%)	3 (2.8%)
**During COVID-19 pandemic, were there challenges in assessing and managing:**				
Physical needs	8 (16.3%)	3 (12.5%)	6 (17.6%)	17 (15.9%)
Psychological needs	14 (28.6%)	6 (25.0%)	9 (26.5%)	29 (27.1%)
Social, family, or carer needs	20 (40.8%)	16 (66.7%)	20 (58.8%)	56 (52.3%)
Spiritual needs	11 (22.4%)	9 (37.5%)	6 (17.6%)	26 (24.3%)
Cultural needs	5 (10.2%)	4 (16.7%)	3 (8.8%)	12 (11.2%)
Person-centered care	7 (14.3%)	4 (16.7%)	6 (17.6%)	17 (15.9%)
**During COVID-19 pandemic, were there challenges managing the following symptoms:**				
Agitation	9 (18.4%)	4 (16.7%)	10 (29.4%)	23 (21.5%)
Breathlessness	9 (18.4%)	2 (8.3%)	8 (23.5%)	19 (17.8%)
Fever/shivering	6 (12.2%)	1 (4.2%)	3 (8.8%)	10 (9.3%)
Cough	8 (16.3%)	1 (4.2%)	3 (8.8%)	12 (11.2%)
Pain	6 (12.2%)	1 (4.2%)	3 (8.8%)	10 (9.3%)
Fatigue	10 (20.4%)	2 (8.3%)	5 (14.7%)	17 (15.9%)
**Do you think the quality of care provided to those approaching the end of life fluctuated during the pandemic?**				
Yes	8 (16.3%)	8 (33.3%)	10 (29.4%)	26 (24.3%)
No	40 (81.6%)	16 (66.7%)	23 (67.5%)	79 (73.8%)
Missing	1 (2.0%)	0 (0.0%)	1 (2.9%)	2 (1.9%)
**During the pandemic, did your care home use any of the following to deliver palliative and end-of-life care:**				
End-of-life programme (e.g., Gold Standards Framework, six steps)	11 (22.4%)	14 (58.3%)	14 (41.2%)	39 (36.4%)
National guidance or policies for palliative and end-of-life care	20 (40.8%)	11 (45.8%)	16 (47.1%)	47 (43.9%)
Local guidance or policies for palliative and end-of-life care	22 (44.9%)	16 (66.7%)	19 (55.9%)	57 (53.3%)
Guidance on symptom control (e.g., breathlessness)	12 (24.5%)	11 (45.8%)	15 (44.1%)	38 (35.5%)
Guidance on communication	7 (14.3%)	6 (25.0%)	7 (20.6%)	20 (18.7%)
Electronic palliative care coordination systems (or equivalent)	5 (10.2%)	5 (20.8%)	3 (8.8%)	13 (12.1%)
Other	8 (16.3%)	2 (8.3%)	6 (17.6%)	16 (15.0%)
**Since the start of the pandemic, are you using telehealth (e.g., telephone, video calls, use of laptops, tablets) more for palliative and end-of-life care?**				
Yes	42 (85.7%)	23 (95.8%)	29 (85.3)	94 (87.9%)
No	6 (12.2%)	1 (4.2%)	3 (8.8%)	10 (9.3%)
Total (missing)	1 (2.0%)	0 (0.0%)	2 (5.9%)	3 (2.8%)
**What are you using telehealth for?**				
Staff education	28 (57.1%)	13 (54.2%)	19 (55.9%)	60 (56.1%)
Communication with healthcare professionals	44 (89.8%)	23 (95.8%)	27 (79.4%)	94 (87.9%)
Communication with families	38 (77.6%)	20 (83.3%)	25 (73.5%)	83 (77.6%)
Assessment/monitoring of residents	22 (44.9%)	15 (62.5%)	20 (58.8%)	57 (53.3%)
Other	0 (0.0%)	0 (0.0%)	4 (11.8%)	4 (3.7%)
**If you need advice about palliative and end-of-life care, who do you usually ask?**				
Specialist palliative care and hospice team	31 (63.3%)	22 (91.7%)	30 (88.2%)	83 (77.6%)
Community nurses	36 (73.5%)	3 (12.5%)	8 (23.5%)	47 (43.9%)
Community pharmacist	14 (28.6%)	4 (16.7%)	4 (11.8%)	22 (20.6%)
Other community services (e.g., therapists)	8 (16.3%)	2 (8.3%)	3 (8.8%)	13 (12.1%)
GPs	36 (73.5%)	19 (79.2%)	27 (79.4%)	82 (76.6%)
Geriatricians	2 (4.1%)	1 (4.2%)	3 (8.8%)	6 (5.6%)
Other	3 (6.1%)	0 (0.0%)	1 (2.9%)	4 (3.7%)

### Theme 1: Relationship-centered care

Relationship-centered care was identified as an important feature of care that was vital for providing high-quality palliative and end-of-life care within care homes. It was characterized by staff creating a sense of home and nurturing close relationships with residents and their families; relationships so strong that they were akin to family bonds and extended beyond residents' deaths:

“*We have a really good rapport with our relatives. One lovely initiative was that as the staff weren't allowed attend the funeral of one of our residents, due to Covid restrictions, the family asked if the funeral cortege could leave from here. That was wonderful, although we all had to socially distance. The hearse drove through in the front of our home and all the residents and staff were able to say goodbye. It was very emotional and a first for us.”* [manager, dual residential and nursing home, survey free-text response]

The pandemic disrupted the ability of care home staff to engage in the activities needed to deliver relationship-centered care. In explaining this, qualitative findings converged with the quantitative data in identifying that COVID-19 particularly disrupted the assessment and management of residents' social needs (56, 52.3%) in addition to their physical (17, 15.9%) and psychological (29, 27.1%) needs. Social distancing and visiting restrictions disrupted relationship-centered care as staff were less able to provide emotional support, reassurance, and physical presence when a resident was dying.

Most care homes (84, 78.5%) allowed families to visit residents who were near the end of life. However, visiting restrictions (especially for residents not thought to be dying) and shifts to virtual forms of communication disrupted the ability of families/friends to engage socially and provide emotional support. This had detrimental consequences for both residents and their families (including in bereavement), alongside having a negative impact on the vibrancy and homely atmosphere that usually characterized care homes:

“*The biggest thing was not having families and friends and volunteers here because the home is very vibrant; we're a really big part of the community, and all of a sudden that didn't happen … We traditionally, up until that time, would have had lots of family involvement … I don't think we can underestimate that, not only from the residents' point of view, but the family's point of view and how they've been able to deal with things afterwards. Because, for some people, it's sort of left them with an unanswered question, or they don't feel that they've come to the end of their journey properly.”* [manager, nursing home, interview]

Alongside social distancing, using personal protective equipment (PPE) further disrupted relationship-centered care; it compromised in-person and non-verbal communication and may have contributed to difficulty assessing and managing residents' psychological (29, 27.1%) and spiritual (26, 24.3%) needs:

“*It was terrible. It was undignified. I would use such a strong word as traumatic for both myself and my staff. It was definitely traumatic for the relatives we know. We don't know how it was for the resident. It was dismal, it was really awful. … the fact that I couldn't care in the way I would like to care. You want to care with someone holding their hands and sitting and playing music and, you know, all of that. There were no frills.”* [manager, residential home, interview]

### Theme 2: Pillars of relationship-centered care

Care home staff adapted rapidly to preserve as many elements of relationship-centered care as possible in order to uphold the quality of the palliative and end-of-life care that they delivered to residents. Most participants (79, 73.8%) reported that the quality of care for residents at the end of life was maintained despite the many challenges. The ability of care homes to adapt and respond to COVID-19 was contingent on different “pillars” of care being present. Each pillar describes essential structures and/or processes of care that were required to preserve relationship-centered care. Inequities within the care home sector meant that in some services these pillars were compromised, and relationship-centered care suffered.

#### Pillar 1: Integration within health and social care systems

A key pillar that supported the ability of care homes to provide relationship-centered palliative and end-of-life care was integration with external health and social care services. Quantitative data highlighted that care homes commonly connected with GPs (82, 76.6%) and specialist palliative care teams (83, 77.6%) for advice on palliative and end-of-life care, while residential homes were more likely to integrate with community nursing teams (36, 73.5%).

Qualitative data provided insight into the variability with which care homes were integrated within local health and social care systems. Poor integration with external services led to care home staff spending hours trying to access (often unhelpful) generic national or local telephone advice:

“*Our major source of stress when dealing directly with deteriorating health and end-of-life care was caused by the interminable wait to be able to access NHS24 out of hours [NHS24–*National Health Service urgent care telephone advice] …* Accessing out-of-hours help and support was outrageously difficult. In my daily incident log, I noted one incident: “Contacted 111* [NHS24 urgent care telephone advice] *for help as two ladies were deteriorating. On phone for 40 min. They disconnected us. On again for 45 min, they disconnected us again. Called again'—after another 2 h and 5 min we got through. Eventually got put through to a doctor. The doctor thought hospital at home would not be an option as it's the holidays, and therefore she would look at the use of the ACP* [advance care planning] *medications. I was able to tell her that, actually, not only was hospital at home working despite the bank holiday, I had a phone number of the consultant in charge' … Why do care home managers not have access to the same “hotline” that pharmacists and others have? Why must we be left at the mercy of 111 and pharmacists … this stress could be removed along with the risk of a resident being needlessly in pain”*. [manager, residential home, survey free-text response]

Qualitative data complemented quantitative data in providing a deeper understanding of the key ingredients needed for integrated working throughout the pandemic. This was conveyed by a care home nurse who summarized that good integration was based on “*excellent relationships through professional networking; mutual trust and respect; professionalism”* [manager, nursing home, survey free-text response].

Integration between services during the pandemic was strengthened by pre-existing and explicit ways of working, alongside robust relationships with external healthcare professionals. Processes that facilitated these relationships were care homes having direct lines of communication, such as a single point of contact, “a hot-line” to access support/advice, and regular case note reviews of residents in multidisciplinary team meetings. Whilst for some care homes these forms of integration existed before the pandemic, others were able to use COVID-19 as a springboard for their development. Participants perceived that these processes helped to prevent hospital admissions, ensure timely access to care and treatments (including medications), improve clinical assessment of residents, facilitate advance care planning and after-death care, and allow care home staff more time to focus on relationship-centered care to meet residents' needs:

“*We had a lot of support from the district nurses. We have a lot of support from the community matrons... We're not like a medical nursing home; we're a residential home. There was a lot of things that we couldn't do without district nurse input. So, I think, like, administering end-of-life drugs and things like that. We have a lot of input from the community matrons, which was so helpful to us.”* [other, residential home, interview]

#### Pillar 2: Digital inclusion for end-of-life care

Almost all (94, 87.9%) care homes reported increased use of remote consultations to provide palliative and end-of-life care. Telehealth was used for communication with healthcare professionals (94, 87.9%) and residents' families (83, 77.6%), staff education (60, 56.1%), and for the assessment and monitoring of residents (57, 53.3%).

Qualitative data illuminated how participants' experiences of and views on using digital resources to support relationship-centered care were affected by existing infrastructure. For some, using digital technology was an effective way of accessing skilled clinical support, preserving connectivity with residents and their families, and supporting integration. For others, there was a lack of basic equipment and/or Wi-Fi, meaning the potential benefits of digital technology could not be harnessed. This was detrimental to the provision of relationship-centered care, as it limited integration and restricted emotional involvement/contact between residents, their families, and staff:

“*We had one lady who was end of life, not from COVID, but was end of life and had been for a little while, and her daughter, before the pandemic, used to come in and read her a book. They would read a book together; they would do a chapter together each time she came in. So, she carried on doing that, but did it with the iPad next to her. And that was really something that was really comforting for her daughter, because she still felt that bit of involvement, although she physically couldn't be there.”* [manager, nursing home, interview]

“*We have atrocious internet here. So, yes, we have used emails, but we're not able to get things like Skype, hence me being on the phone… I think the lack of Skype, etc., did impact on the emotional support for be it staff, residents, and relatives. You know, the emotional contact between relatives and their loved ones.”* [deputy manager, nursing home, interview]

While many recognized the potential value of using digital technology in facilitating palliative and end-of-life care, others voiced concerns that it was difficult and distressing for some patients to use (especially those with dementia), alongside sometimes detracting from important in-person elements of relationship-centered care:

“*… people with dementia, there wasn't a void for them particularly, because it was filled with something else. They didn't always get the face timing and the video calls. So, sometimes we had to make a bit of a call on that to say, actually, it's really distressing, because they don't know why you're not there, although you appear to be there.”* [manager, nursing home, interview]

#### Pillar 3: Workforce support

The third pillar that impacted the ability of care homes to provide palliative and end-of-life care was having a sufficiently supported workforce. This meant staff having the capacity to provide relationship-centered care with sufficient time and both practical and emotional support. Staff shortages (48, 45.3%), stress (75, 70.1%), and absenteeism (46, 43%) had a cumulative effect on the ability of staff to provide care. Sickness and shielding/isolation resulted in increased workloads placed on smaller numbers of staff. This had physical and psychological impacts on staff, meaning that they had less capacity to deliver palliative and end-of-life care to the standard that they wanted:

“*Because our workload had increased due [to] other residents being sick at the same time, we weren't able to give the level of care to the residents that we would normally give during their last hours of life … you're not giving your normal five-star service, and you know that, and you know that person is at their end of life, but there are other people who need your assistance – and because we were short of staff, because they were off either isolating, household isolating, self-isolating”*. [manager, nursing home, interview]

The survey data showed that 45.8% of care homes hired agency staff to support their workforce. Qualitative data expanded on this by highlighting participants' concerns that use of agency staff could impair relationship-centered care for people approaching the end of life because of having more limited skills in end-of-life care, as well as insufficient knowledge of residents and their preferences:

“*We had up to 50 staff off sick or shielding at one time. Only essential care was the priority due to staff numbers. Agency staff were employed, though these rarely turned up. This all impacted end-of-life care—the ability to closely monitor residents and react appropriately…. The home was using staff who were not familiar with the residents, unlike our own staff who knew their plans for end of life and would respond to palliative concerns”*. [manager, dual residential and nursing home, survey free-text response]

These workforce pressures occurred in a context of an “infodemic”. After an initial dearth of information, care home staff felt inundated by information that was confusing and changed rapidly. The time it took to make sense of, disseminate, and act on this meant that the staff's focus shifted away from relationship-centered care toward more logistical/administrative duties and maintenance of essential care:

“*Care home staff have had a horrendous time over the last 15 months … as the pandemic continued, we were inundated with documentation issuing directives and guidance from so many bodies it became overwhelming and still is. The information to be disseminated needs to be summarized and bullet pointed for ease of access and legibility, especially for small care homes and providers who do not have a huge HR department to sieve through and highlight the most relevant requirements”*. [manager, dual residential and nursing home, survey free-text response]

### Theme 3: Parity of esteem

Parity of esteem captures the societal and economic foundations that are essential for supporting the different pillars of care needed for high-quality relationship-centered care. Despite making significant contributions to providing palliative and end-of-life care throughout the pandemic, participants felt their efforts and expertise went unrecognized and were undervalued by the government, media, public, and wider healthcare system.

There was a common misperception that care homes served as a “*dumping ground for old people who are not able to do things for themselves”* [manager, nursing home, interview] or a place where other settings of care could discharge people testing positive for COVID-19. Many felt excluded from national gestures (i.e., public clapping for the National Health Service) and positive media stories regarding the work of the NHS, which affected the morale of an already stretched and tired workforce:

“*Staff got really tired because they were doing really long shifts and not wanting to leave staff at the care home, because they were short of staff as well … And it was horrendous. It was distressing. Even though the work they've done is unbelievable, I don't think the government or society has particularly placed any massive accolade on the work that the care home staff have done, who are predominantly untrained healthcare assistants. And I think they don't feel that valued, and I think that contributes to poor mental health. They've done a brilliant job.”* [registered nurse, nursing home, interview]

While many participants perceived a lack of media support during the pandemic, others felt the media actively targeted, blamed, and spread misinformation about care homes in relation to COVID-related deaths:

“*I've also got to say, the NHS staff were lauded as if they were supernatural beings. They were angels and heroes and all that. And then you've got the health and social care staff, and stories starting to appear in your daily rags about scandals at nursing homes. We had reporters at the front, guys with cameras, and guys trying to speak, trying to locate relatives [in incidents where residents] had died to try and get dirt on the place, you know? Aye, it happened. So, then you look at an article, and it's actually about your place and about people in the wider MDT that you're working with that you know, for a fact, have done their absolutely utmost, and the thing would have been twice as bad if you didn't have them. And their actual practices are getting brought into question by somebody that can't put a few paragraphs together in a way that makes journalistic sense… That's in print.”* [deputy manager, nursing home, interview]

This disparity of esteem had tangible impacts on the ability of care home staff to provide palliative and end-of-life care safely and confidently throughout the pandemic. Some participants felt as though care homes had been deprioritised or abandoned by government authorities and health services in the distribution of adequate PPE and testing kits:

“*I don't think that the health authority phoned us once… The health service was not supportive as such, and it was upsetting when we'd phone our regular suppliers for PPE or things like that and we're being told: ‘Oh, it has to be directed to the NHS', like we were nothing. And you just felt that care homes were kind of left out in the cold.”* [manager, nursing home, interview]

## Discussion

The aims of this study were to investigate the response of UK care homes in meeting the rapidly increasing need for palliative and end-of-life care during the COVID-19 pandemic and to propose recommendations for strengthening the provision of palliative and end-of-life care within care homes. The novelty and contributions of this study lie in the adoption of a mixed methods approach, in which we triangulated closed-ended and free-text survey responses with in-depth qualitative interview data. As such, this study contributes to a more thorough and contextualized understanding of how and why the delivery of palliative and end-of-life care in care homes was affected by the COVID-19 pandemic, and how policy changes may support its provision both now and in the future.

The findings demonstrate that relationship-centered care is crucial to high-quality palliative and end-of-life care within care homes. Relationship-centered care is already a well-established concept in the health and social care literature, referring to how the quality of relationships between residents, their families, and practitioners influences processes and outcomes of care (32). Despite the disruptions to relationship-centered care (predominantly due to visiting restrictions and social distancing policies), the majority of participants in this study reported that the quality of care for residents at the end of life was maintained. These findings contrast with recent research conducted with bereaved relatives, in which studies found that those who died in care homes during the COVID-19 pandemic were more likely to experience poorer outcomes before death compared to other settings of care (e.g., hospice, home, and hospital) (18, 21). These differences may be explained by the extent to which the “pillars of relationship-centered care” were present across services and how this affected the ability of care homes to uphold the provision of high-quality palliative and end-of-life care in the context COVID-19.

One of these pillars was good integration with health and social care services. Previous studies have highlighted the importance of integration within health and social care (20, 33) and palliative and end-of-life care (34). Care home-specific research has also demonstrated that services that provide “wraparound care” that is not reliant on single practitioners and value care homes as partners in the care of older people are more likely to lead to improved resident and system outcomes (35, 36). Our findings highlight how care home staff drew on “meso” forms of integration (34) to support high-quality palliative and end-of-life care through creating new (or strengthening already existing) connections with primary, specialist palliative, and community care services. Previous research has identified “key ingredients” for successful integrated healthcare (33, 34). Our findings extend these by showing the importance of mutual trust, a sense of partnership, and strong community ties underpinning integration between care homes and external services. They also show that inequities in integration existed, which impacted on patient care. Whilst some care home services already had well-established integration with external services, and others were able to forge these networks during the pandemic, others did not. Instead, some services relied on generic, slow, and unhelpful advice for residents at the end of life, impacting the quality of care that they were able to deliver. Future research, resources, and policy changes are needed to address this and understand how to optimize integration between care homes and external services to support the provision of high-quality palliative and end-of-life care in care homes.

Our study demonstrates how the “digital divide” was another pillar of care that impacted the ability of care homes to harness the potential benefits of digital technology in the provision of relationship-centered care. In line with previous work (37), where the necessary infrastructure and equipment was accessible and used effectively, this could preserve some elements of relationship-centered care and facilitate integration with external services. However, these were not always in place, and participants did not always feel supported or able to work in these ways. Moreover, video consultations could sometimes be difficult to use, distressing and confusing for some patients (especially those with cognitive impairments), and detract from important elements of relationship-centered care. Careful consideration of these issues—including a more systematic evaluation of the benefits and limitations of digital technology in this context (37)—is needed (alongside policy and service developments) to embed digital technology within care homes in ways that optimize relationship-centered care.

This study supports previous research that highlights the key role that care home managers play in supporting their team to deliver high-quality palliative and end-of-life care, but it also highlights how the pressures of the pandemic had profound physical and psychological impacts on staff that undermined their ability to do this (9, 10, 20). These impacts were cumulative and disrupted the capacity of care home staff to deliver care to the standards that they wanted. The gradual accumulation of physical, emotional, and psychological distress experienced by healthcare professionals throughout the pandemic has been well-documented, including across palliative care settings (38–40). These experiences are situated within a workforce crisis in the UK adult health and social care sector, characterized by low pay, high staff turnover, and difficulty in recruiting and retaining staff (41). The recruitment and retainment of skilled staff within care homes relies on them feeling valued and adequately supported in the delivery of care (including palliative and end-of-life). This includes providing care home managers with adequate emotional and practical support so that they can support their teams, alongside ensuring care home staff have sufficient resources and time to deliver high-quality care. Moreover, it is important that the burden and pressures of developing these support strategies and solutions are not placed solely on individuals. Governments and organizations have a duty of care and ethical responsibility to mitigate workforce shortages, maintain staff wellbeing, and ensure that adequate structures and processes of care are in place to support staff and services in the delivery of high-quality palliative and end-of-life care in care homes (38, 42).

However, despite the extensive contribution of care homes in meeting the rapid rise in the need for palliative and end-of-life care throughout the pandemic, participants felt that their role was often unrecognized and undervalued. This had tangible impacts on the provision of palliative and end-of-life care, including not being prioritized in the distribution of PPE or testing kits. Rather than providing them with the support and resources needed, there was a prevailing sense among practitioners that the government in England failed to protect their (and their patients') health throughout the pandemic (43), with a High Court judgement concluding that insufficient equipment was the result of unlawful policy decisions by the government (44).

### Implications for policy

During the first weeks of the pandemic, care homes became the most common place of death in England, reaching the levels projected for 2040 (2, 3). The COVID-19 pandemic, therefore, has provided a “stress test” in which lessons can and should be learned for the future provision of palliative and end-of-life care in care homes. In contributing to this, we identify key policy priorities for strengthening these provisions (see Table 3). These priorities should be considered by policymakers to ensure that care homes are equipped with the resources, capacity, and expertise needed to deliver high-quality palliative and end-of-life care, and ameliorate serious health-related suffering for an aging population with an increasing prevalence of frailty, dementia, and multimorbidity globally (45).

**Table 3:** Policy priorities for the provision of palliative and end-of-life care in care homes.

**Policy priorities**	**Policy implications**	**Ways to action this**
1. Integration within health and social care systems	**‘Spirit of partnership': integration with health and social care services**: Care homes should be well-integrated with external services so that they can receive timely access to support and specialist advice that helps with the delivery of palliative and end-of-life care. This may be through strengthening already established links across the health and social care sector or creating new ones.	•Palliative care representation within multidisciplinary team meetings in care homes to provide specialist advice that supports decision-making. •Universal representation of care homes within local governance systems (such as integrated care systems in England).
2. Digital inclusion for end-of-life care	**Digital inclusivity** An equity-centered approach to future digital policies—in which care homes keep up with technological advancements in healthcare—is crucial. This must include ensuring that all care homes have access to the resources (including equipment and infrastructure) needed to tap into the potentials of digital ways of working to support palliative and end-of-life care.	•Including care homes in national digital healthcare strategies and agreeing on a minimal level of I.T. support, training, and infrastructure that is needed to support palliative and end-of-life care in care homes. •Supporting integrated working through putting interoperable electronic systems (e.g., electronic patient records that can be easily shared and accessed across services and settings) at the heart of any digital healthcare policy/strategy.
3. Workforce development	**Workforce development, training, and support in delivering palliative and end-of-life care:** Staff working in care homes should feel confident, skilled, and supported in the delivery of palliative and end-of-life care, including assessing and managing physical, psychosocial, spiritual, and cultural needs. Training in palliative and end-of-life care should be accessible and tailored to care home staff.	•To address the social care workforce crisis, career pathways and opportunities must be created that attract and retain staff into care home roles, including graduate nurses and healthcare assistants. Better conditions of work for all, including pay and continuing professional development funding, is also essential.
4. Support for care home managers	**Support for care home managers**: Care home managers play an integral role in supporting their teams to deliver high-quality palliative and end-of-life care. Ensuring that care home managers have adequate emotional and practical support to do this is crucial.	•Initiatives that foster collaborations between care home managers, alongside creating alliances with hospices, may better support care home managers through facilitating the sharing of best practice, skills and capabilities, knowledge, support, and advice. Care home managers should also be included in the development of policy and guidance that directly affect the sector.
5. Address (dis)parity of esteem	**Valuing the role of care homes and care home staff:** Care homes and their staff play a key role in the delivery of high-quality palliative and end-of-life care both during and outside of pandemics. These contributions should be recognized and valued by the government, media, public, and wider healthcare system.	•Within policy and planning, care homes should be positioned as equal partners within the health and social care system through better funding, staffing, and representation to influence policy decision-making at local, regional, and national levels.

The policy recommendations suggested here inform, extend, and align with already existing policies and initiatives. Internationally, this includes the World Health Assembly 2014 declaration on strengthening the integration of palliative care as an essential service within universal health coverage (46), and the Worldwide Hospice Palliative Care Alliance call to build integrated palliative care programmes and services, including in care homes (47). Within the UK, the NHS England 2019 Long Term Plan, which includes the Framework for Enhanced Health in Care Homes (48), is a policy example pursuing integrated approaches that prioritize care centered on individual residents, their families, and care staff, with care needs met through a whole-system, collaborative approach.

### Strengths and limitations

Through combining survey and qualitative interview data over different time points, this study provides an in-depth understanding of how COVID-19 affected the provision of palliative and end-of-life care in care homes. Survey respondents and interview participants were sampled across care home size, type, and region, enhancing the generalisability of the findings. Whilst the survey was only offered in the English language and distributed to care homes within the UK, we anticipate that many of these findings, alongside their policy implications, also resonate with and have relevance to care home/long-term residential facilities internationally.

We recognize that a limitation of this study is that, despite the effort to maximize recruitment for the survey, the final sample is relatively small. We sought to maximize the data by purposively over-recruiting in the qualitative interviews, targeting areas of underrepresentation in the survey, such as smaller care homes. Another limitation of this study is that survey and interview data predominantly represent the voices/perspectives of care home managers. These may not accurately reflect the views of other professionals/staff (e.g., nurses, healthcare assistants, and care workers/assistants) who provided palliative and end-of-life care within these organizations throughout the pandemic. Moreover, beyond the input from our Patient and Public Involvement group, the perspectives of the residents and their families/carers were not included in this study. Future research focused on these perspectives will help to further our understanding of the impact of COVID-19 on the provision of palliative and end-of-life care in care homes, and how we can learn from these to better practice in the future.

## Conclusion

This study not only highlights the vital role of relationship-centered care when providing palliative and end-of-life care in care homes, but also how this aspect of care was disrupted during the COVID-19 pandemic due to visiting restrictions, social distancing measures, and staff shortages. The ability of care homes to adapt and provide relationship-centered care was dependent on different “pillars” of care being present. These included integrated working with health and social care providers, having access to the equipment and infrastructure needed to take advantage of digital ways of working, and feeling practically and emotionally supported. Although care home staff made significant contributions in providing palliative and end-of-life care during the pandemic, they felt that these went unnoticed and undervalued. These findings inform key policy priorities that should be considered by policymakers to ensure that care homes are equipped with the resources, capacity, and expertise needed to deliver palliative and end-of-life care both now and in the future. This is especially relevant given the known, escalating need for this type of care in care homes over the next 20 years.

## Data availability statement

Applications for use of the survey data can be made for up to 10 years and will be considered on a case-by-case basis on receipt of a methodological sound proposal to achieve aims in line with the original protocol. The study protocol is available on request. All requests for data access should be addressed to the Chief Investigator via the details on the CovPall website (https://www.kcl.ac.uk/cicelysaunders/research/evaluating/covpall-study/covpall-care-homes and palliativecare@kcl.ac.uk) and will be reviewed by the Study Steering Group.

## Ethics statement

Institutional ethical approval was granted by King's College London Research Ethics Committee (LRS-19/20-18541). The patients/participants provided their written informed consent to participate in this study.

## Author contributions

KS and CE are grant holders, joint chief investigators, and were responsible for study conceptualization and development of the study protocol, with critical input from grant co-applicants. IH, CG, SB, and CE-S are co-applicants for funding. IT and IB co-ordinated data collection with the assistance of KS and CE-S. Data analysis was led by SO and ABr. ABr, KS, and CE drafted the original manuscript. All authors had access to all study data, discussed the interpretation of findings, take responsibility for data integrity and analysis, contributed to the analysis plan, and provided critical revision of the manuscript for important intellectual content.
