# Supplementary material for: Provision of palliative and end-of-life care in UK care homes during the COVID-19 pandemic: A mixed methods observational study with implications for policy
Source: Front Public Health. 2023 Mar 14;11:1058736. doi: 10.3389/fpubh.2023.1058736 (PMC10043445; doi:10.3389/fpubh.2023.1058736)
Supplement: Supplementary file 1 [file Data_Sheet_1.PDF]

**Table of contents**

**S.1. CovPall Care Homes Survey ……………………………………………………………….. 2**

**S.2. CovPall Care Homes Interview Topic Guide …………………………………………… 14**

## S.1. CovPall Care Homes Survey

## Exploring provision of palliative and end-of-life care in care homes during the COVID-19 pandemic

Thank you for agreeing to complete this survey. We are trying to find out about how palliative and end-of-life care was provided in care homes during the COVID-19 pandemic, what the main challenges were and how care homes managed to overcome them.

COVID-19 has had a devastating impact on care homes, their residents, families and staff. Many care home residents in England have died from COVID-19, and many more have experienced distressing symptoms. Palliative and end-of-life care can alleviate symptoms and improve family bereavement. However, providing high-quality palliative and end-of-life care in care homes has been particularly challenging during COVID-19. Your participation will help us understand the response of care homes to COVID-19 including experiences, preparedness and impact on the care home workforce.

We realise you are very busy right now, so we have tried to balance collecting the information that patients, policy makers and services think is most helpful, with keeping the questionnaire as short as we can.

The questionnaire has 8 sections, and should take no longer than 30 minutes to complete, although it may depend on how much additional/open comments you wish to share. We will consider everything that you say. Although the grouped results will be shared, we will not name your unit unless you ask us to.

You can pause the questionnaire by clicking the "Save & Return Later" button at the bottom of each page. You will be given a code to enable you to continue later – please remember this code. There should be a "returning" option at the top right of this page. Please write this code down. If you forget the code or wish to correct errors after you have clicked "Submit", please email palliativecare@kcl.ac.uk with CovPall_CareHome Survey in the subject line.

If you would like information or help with completing the survey, or would prefer someone to read the questions to you while you give the answers over the telephone / zoom call, please send your contact details to palliativecare@kcl.ac.uk with CovPall_CareHome Survey in the subject line.

If you have any concerns about this questionnaire or this study, please email katherine.sleeman@kcl.ac.uk.
CovPall_CareHome is led by Professor Katherine Sleeman and Dr Catherine Evans of the Cicely Saunders Institute, King's College London, with a team of partners from different organisations and backgrounds. Care home representatives and policy makers have inputted into this questionnaire and our plans. For more information see https://www.kcl.ac.uk/cicelysaunders.

This study has been granted ethical approval by: Research Ethics Committee, King's College London (Ref No: LRS-19/20-18541). Completion of this survey is indication of your consent to take part in this research study.

Please tick all responses that apply. Some questions in Section 1 and all questions in Section 3 are required.

For all questions in the survey, please reflect on your experiences since March 2020, unless stated otherwise. We are particularly interested in how your experiences have changed over the COVID-19 pandemic.

### 1. ABOUT YOU

1.1 Care Home Name * ______________________

1.2 Name of the person completing the survey ______________________

1.3 Contact email * ______________________

(We need this information so we can help you get back into the survey, or help you to complete it, if you have difficulty)

1.4 Phone number

___________________________________

1.5 Care home postcode *

_______________________________

1.6 Your role *

- ☐ Manager
- ☐ Deputy Manager
- ☐ Registered Nurse
- ☐ Senior Carer
- ☐ Team Leader
- ☐ Other (a box will open)

Other role *

_______________________________

## 2. ABOUT YOUR SERVICE

2.1 Please describe your care home registration

- ○ Residential Care
- ○ Nursing care
- ○ Both residential care and nursing care
- ○ Other (a box will open)

Other (please state)

_______________________________

2.2 Who is your care home run by

- ○ Charity
- ○ Private company
- ○ Private not-for-profit company
- ○ Local Authority
- ○ Other organisation type (a box will open)

Other organisation type (please state)

___________________________________

2.3 How many beds are there in your care home

___________________________________

2.4 On average, what percentage of residents are funded by a local authority

___________________________________

(Give a number 0-100 (without a % sign))

2.5 On average, what percentage of residents are funded by Continuing Healthcare funding (CHC)

___________________________________

(Give a number 0-100 (without a % sign))

2.6 On average, what percentage of residents are from black or minority ethnicity groups

___________________________________

(Give a number 0-100 (without a % sign))

2.7 On average, what percentage of staff are from black or minority ethnicity groups

___________________________________

(Give a number 0-100 (without a % sign))

## 3. STAFF CONFIDENCE IN PROVIDING END OF LIFE CARE

Please rate your degree of confidence with the following topics relating to provision of palliative and end of life care

| | Need further basic instruction | Confident to perform with close supervision/coaching | Confident to perform with minimal consultation | Confident to perform independently |
|---|---|---|---|---|
| Answering residents' questions about the dying process * | ○ | ○ | ○ | ○ |
| Supporting the resident or family member when they become upset * | ○ | ○ | ○ | ○ |
| Informing people of the support services available * | ○ | ○ | ○ | ○ |
| Discussing different environmental options (e.g. hospital, home, family) * | ○ | ○ | ○ | ○ |
| Discussing a resident's wishes for after their death * | ○ | ○ | ○ | ○ |
| Answering queries about the effects of certain medications * | ○ | ○ | ○ | ○ |
| Reacting to reports of pain from the resident * | ○ | ○ | ○ | ○ |
| Reacting to and coping with terminal delirium * | ○ | ○ | ○ | ○ |
| Reacting to and coping with terminal dyspnoea (breathlessness) * | ○ | ○ | ○ | ○ |
| Reacting to and coping with nausea/vomiting * | ○ | ○ | ○ | ○ |
| Reacting to and coping with reports of constipation * | ○ | ○ | ○ | ○ |
| Reacting to and coping with limited resident decision-making capacity * | ○ | ○ | ○ | ○ |

## 4. IMPACT OF COVID-19

4.1 Has your care home experienced a confirmed or suspected outbreak of COVID-19 since March 2020 ○ Yes ○ No

(An outbreak of COVID-19 is defined as two or more residents (in a 14-day period) diagnosed with confirmed or suspected COVID-19)

How many suspected or confirmed outbreaks has your care home experienced since March 2020

______________________________

In which month did the first outbreak occur

______________________________

(e.g. Apr 2020)

4.2 Did you experience staff shortages during the pandemic ○ Yes ○ No

How did staff shortages impact on provision of palliative and end of life care in your care home

4.3 During the COVID-19 pandemic, did staff responsibilities in providing care for people nearing the end of life change ○ Yes ○ No

Please tell us more about these changes

4.4 During COVID-19, were there issues with

☐ Staff turnover
☐ Staff redeployment
☐ Staff absenteeism
☐ Staff stress
☐ Staff suffering losses
(check all that apply)

Please describe these issues in more detail and how they affected the delivery of care for people approaching the end of life

Tell us about ways in which you promoted workforce resilience and wellbeing during the pandemic

4.5 During the COVID-19 pandemic, have there been changes in how often you used agency staff ○ Yes ○ No

How have these changes affected palliative and end of life care in your care home

## 5. CARE FOR PEOPLE WITH COVID-19 AND OTHER CONDITIONS APPROACHING THE END OF LIFE

In this section, please reflect on your experiences of caring for residents with and without COVID-19 since March 2020

5.1 During the COVID-19 pandemic, did your care home experience shortages of key medicines for palliative and end of life care

○ Yes
○ No

Please tell us more about these shortages and how they affected care for people approaching the end of life

5.2 During the COVID-19 pandemic, did your care home Yes experience
shortages of equipment or essential No supplies for palliative and end of life
care (e.g. syringe pumps, access to oxygen)

Please tell us more about these shortages and how they affected care for people approaching the end of life

| | |
|---|---|
| 5.3 During the COVID-19 pandemic, did staff in your care home experience challenges in recognising that residents may be dying, including those with COVID-19 and other conditions | ◯ Yes<br>◯ No |

Please tell us more about the challenges experienced in recognising that residents may be dying during the COVID-19 pandemic

| | |
|---|---|
| 5.4 During the COVID-19 pandemic, did staff in your care home experience challenges in assessing and managing people who were approaching the end of life, people who had COVID-19 and those with other conditions | ◯ Yes<br>◯ No<br>((no follow-on question)) including |
| 5.5 During the COVID-19 pandemic, did staff in your care home experience challenges in assessing and managing | ☐ Physical needs<br>☐ Psychological needs<br>☐ Social, family or carer needs<br>☐ Spiritual needs<br>☐ Cultural needs<br>☐ Person-centred care<br>☐<br>(check all that apply) |

Please tell us more about the challenges experienced regarding the assessment and management of people near the end of life

Please tell us how your care home addressed these challenges

| | |
|---|---|
| 5.6 During the COVID-19 pandemic, did staff in your care home experience challenges managing the following symptoms | ☐ Agitation<br>☐ Breathlessness<br>☐ Fever/Shivering<br>☐ Cough<br>☐ Pain<br>☐ Fatigue<br>☐<br>(check all that apply) |

Please tell us about the challenges experienced with control of symptoms

| | |
|---|---|
| 5.7 Do you think the quality of care provided to those approaching the end of life fluctuated during the pandemic | ○ Yes<br>○ No |

How did the quality of provision of palliative and end of life care fluctuate during the pandemic, and what may have influenced this

| | |
|---|---|
| 5.8 During the pandemic, were there changes in practice or innovation in how you cared for people who were near the end of life | ○ Yes<br>○ No |

What were the most successful changes

What made this change/innovation possible

| | |
|---|---|
| 5.9 Did you have residents from ethnic minority populations with COVID-19 | ○ Yes<br>○ No |

Were there any particular situations that were challenging in providing palliative and end of life care to these populations

| | |
|---|---|
| 5.10 During the COVID-19 pandemic, were there challenges in providing bereavement support to relatives after death of a resident | ○ Yes<br>○ No |

What were the challenges and how were they addressed

5.11 Were there any differences or challenges in providing bereavement care to people from ethnic minority groups

○ Yes
○ No

What were the differences and/or challenges

5.12 During COVID-19, were there challenges associated with communicating with families when residents' health deteriorated and/or when they were close to dying

○ Yes
○ No

What kind of challenges were there

How did you address these challenges

5.13 Did your care home allow visitors during the COVID-19 pandemic for people approaching the end of life

○ Yes
○ No

How was the end of life defined in this context

What were the main challenges your care home experienced around visitors for people approaching the end of life. How did you address these

For any remaining challenges, what additional guidance or support may help you to address them in the future

## 6. PALLIATIVE AND END OF LIFE CARE DELIVERY WITHIN CARE HOME

| | |
|---|---|
| 6.1 During the pandemic, did your care home use any of the following to deliver palliative and end of life care | ☐ End of life programme (e.g. Gold Standards Framework, Six Steps, etc.)<br>☐ National guidance or policies for palliative and end of life care<br>☐ Local guidance or policies for palliative and end of life care<br>☐ Guidance on symptom control, e.g. breathlessness<br>☐ Guidance on communication<br>☐ Electronic Palliative Care Coordination Systems (or equivalent, e.g. Coordinate My Care)<br>☐ Other (a box will open)<br>☐<br>(check all that apply) |

Other guidance etc.

________________________________________

Which programmes or policies were most helpful in provision of palliative and end of life care. Was there anything that would have been more helpful

| | |
|---|---|
| 6.2 During the pandemic, were there any challenges in after-death care (e.g. certification of deaths, awaiting removal of body, liaising with undertakers) | ○ Yes<br>○ No |

Please describe the challenges in after-death care in your care home during the pandemic

| | |
|---|---|
| 6.3 Since the start of the pandemic, are you using telehealth more (e.g. telephone, video calls, use of laptops, tablets) for palliative and end of life care | ○ Yes<br>○ No<br>((no follow-on question)) |
| 6.4 What are you using telehealth for | ☐ Staff education<br>☐ Communication with healthcare professionals<br>☐ Communication with families<br>☐ Assessment/monitoring of residents<br>☐ Other (a box will open)<br>(check all that apply) |

Other

________________________________________

6.5 What worked and did not work well with telehealth

## 7. ADVICE AND SUPPORT FROM EXTERNAL SERVICES

7.1 If you need advice about palliative and end of life care, who do you usually ask

- ☐ Specialist palliative care or hospice team
- ☐ Community nurses
- ☐ Community pharmacists
- ☐ Other community services (e.g. therapists)
- ☐ GPs
- ☐ Geriatricians
- ☐ Other (a box will open)

(check all that apply)

Other sources of advice

______________________________

7.2 How easy do you find it to get advice from other healthcare services in your area

| | very difficult | quite difficult | quite easy | very easy |
|---|---|---|---|---|
| Specialist palliative care or hospice team | ○ | ○ | ○ | ○ |
| Community nurses | ○ | ○ | ○ | ○ |
| Community pharmacists | ○ | ○ | ○ | ○ |
| Other community services (e.g. therapists) | ○ | ○ | ○ | ○ |
| Primary Care/GPs | ○ | ○ | ○ | ○ |
| Geriatricians | ○ | ○ | ○ | ○ |

7.3 What helps your care home integrate with external services such as primary care, community or palliative care teams

During COVID-19

7.4 Were there difficulties in accessing help from someone coming into your care home when you needed it (e.g. GPs, community nurses)

- ○ Yes
- ○ No

Please tell us how this affected palliative and end of life care provision in your care home

__________________________________________

7.5 Were multidisciplinary team meetings (MDTs) held to discuss care for people approaching the end of life

- ○ Yes
- ○ No

Who attended these meetings

__________________________________________

| | very difficult | quite difficult | quite easy | very easy |
|---|---|---|---|---|
| 7.6 How easy did you find it to get advice about palliative and end of life care | ○ | ○ | ○ | ○ |

| | |
|---|---|
| 7.7 Could you always access palliative and end of life care advice when you needed it | ◯ Yes<br>◯ No<br>((no follow-on question)) |
| 7.8 Did you experience difficulties with clinical decision making around palliative and end of life | ◯ Yes<br>◯ No |

What were the main difficulties or challenges you experienced with decision making around palliative and end of life care

| | |
|---|---|
| 7.9 Have volunteering activities in your care home changed during the pandemic | ◯ Yes<br>◯ No |

How has this change affected palliative and end of life care in your care home

| | |
|---|---|
| 7.10 Were there challenges in your care home regarding treatment escalation plans or advance care planning | ◯ Yes<br>◯ No |

Please tell us more about these challenges and what you did to address them

| | |
|---|---|
| 7.11 Did you have challenges around the process of DNA CPR (do not attempt cardio-pulmonary resuscitation) | ◯ Yes<br>◯ No |

Please describe the challenges that you experienced around the process of DNA CPR

Further Comments

Is there anything else that you would like to tell us about provision of palliative and end of life care during the pandemic, what helped provide good care, and what made this harder

Any additional comments

## 8. FINALLY

8.1 Please indicate if you would like to be contacted regarding any of the following (Tick all that apply)

- ☐ To receive copy of the early reports and our newsletters as the findings emerge
- ☐ For us to check any information with you
- ☐ To be acknowledged as responding to this questionnaire (listed along with other services) in the reports and any publications
- ☐ To participate in an interview asking more in-depth questions about your experiences during the pandemic, for which you will receive a £20 voucher

8.2 Would you like to be entered into a prize draw to receive a £100 voucher of your choice

○ Yes
○ No

8.3 If you wish us to use a different Name or Email for the above (instead of the ones already given) please specify here

________________________________________

8.4 All results from the survey will be anonymised before publication. If you would like your care home to be mentioned and acknowledged in our reports, please tell us how we should refer to it here

________________________________________

You will be free to opt out of receiving the updates at any time, your details will not be passed onto other organisations or used for anything other than with your explicit consent above

Your individual responses will remain confidential, they will be analysed pseudonymously by the research team, with your service identified only by a code number unless you explicitly ask us to do otherwise

Thank you for your help at this difficult time

PLEASE NOW CLICK SUBMIT

You must click "Submit" or "Save and Return Later" to save any data you have entered

## S.2. CovPall Care Homes Interview Topic Guide

### CovPall_CareHomes

### Qualitative Interview Topic Guide

**Note:** Aim of the interview is to explore more in-depth challenges and facilitators to providing palliative and end-of-life care for residents during the COVID-19 pandemic, and make recommendations for policy

**Introduction**

Thank you for agreeing to take part in an interview for the CovPall_CareHomes study. We are interviewing people to understand

a) Their experiences of providing palliative and end-of-life care during the COVID-19 pandemic
b) What were the challenges and facilitators to delivering palliative and end of life care
c) What are the key lessons learnt during the pandemic to sustain delivery of high-quality care

**Consent**

- Review the information sheet and opportunity to ask questions. Talk through
    - Understand the purpose of the study
    - The interview will be audio-recorded
    - The recording is typed up, but all names or places that could identify you, or other people or places are removed. Once we have typed the interview, the recording is destroyed.
    - We may publish quotes from your interview, but we use an identification number and don't include any names or details of places to ensure you cannot be identified.
    - Do you have any questions before we start?
- Ask if happy for the consent for the interview to be recorded

**Start consent audio recording**

- Talk through each item on the consent form, and confirm yes or no response for each item, and mark and initial the response on the informed consent
- Ensure participant name is on the consent form and the date.
- Sign and date the consent form as the person completing the consent, and indicate for the person's signature consent digitally recorded and date
- Ask the participant if they would like to receive a copy of the consent form, which you can email or post to them.
- Reiterate that the interview is going to be anonymous, we won't share the name of the care home or individual when we report the data, and their views/experiences won't be linked to their name or care home in any of the reports

**Stop recording once consent is complete**

Are you happy for me to start the interview and to turn on audio recorder?

Please remember that if you wish to stop the interview at any point just signal to me (agree e.g, raising hand).

**Start new audio data file for the interview**

**1) Delivering palliative and end of life care**

To begin, can you talk me through what it has been like providing palliative and end of life care during the COVID-19 pandemic?

- What facilitated you to deliver palliative care?
  - PROMPT And end of life care
- Did your care home have a higher proportion of deaths during the pandemic than usual?
- What challenges did you experience?
- How did you manage these challenges?

**2) Guidance and resources**

- Can you tell me about, what informed how you provided palliative and end-of-life care in the care home during the pandemic?
- How helpful did you find using [this guidance/resource – use name, explore each in turn]?
  - PROMPT: Name(s) of [guidance/resource/policy] used, like Gold Standards Framework for Care Homes
  - PROBE: When did you start using this [guidance(s) – use name]?
  - PROBE: Can you tell me a little bit more about how you used it?
  - PROMPT: What difference did it make working in this way? What was less helpful?
- Can you tell me a little more about, What Covid specific guidance or resources on palliative and end life care you drew upon?
    - PROBE: Can you tell me about how you used it? [explore in turn each guidance, policy, resource]
    - PROMPT: What difference did it make working in this way? What would you have liked more of? What was less helpful?

**3) Symptom treatment and providing comfort**

**Can you tell me about managing symptoms and ensuring comfort, particularly towards the end of life**

- What enabled you to provide optimal treatment of symptoms and comfort?
  - What policies or guidance were helpful in ensuring optimal symptom management?
    - Such as, reuse of medication, anticipatory prescribing
  - What challenges were there around managing physical symptoms, such as when symptoms like breathlessness or agitation caused distress for the person?
  - How did you manage sudden changes leading to rapid deterioration and increasing symptoms and distress?

**4) Person-centred care, decision-making and communication with the family**

- Can you talk me through, what it was like providing person-centred care, like focusing on ‘what mattered to the person’ during the pandemic and the restrictions in place?
  - PROBE: What helped you?
  - PROMPT: For ‘new’ residents who moved to the care home
  - PROMPT: For residents with dementia or cognitive impairment?
- What were the challenges in involving family in making decisions about the person’s care?
- What enabled you to involve the family?

**5) Care goals and advance care planning**

- What changes or innovations were there in setting care goals and advance care planning?
  - What policies were helpful in setting care goals and advance care planning?
  - How did this impact on residents’ care?

**6) Family involvement**

**Can I explore a little more about how you managed government guidance on restricting visiting**

- How did your care home interpret the guidance around visiting restrictions?
  - PROMPT: What was the care home’s policy? How did this change overtime?
  - How did these restrictions impact on providing palliative and end-of-life care for your residents?
  - Can you tell me a little more about, how you managed the balance between allowing family and friends to visit at the end of life and controlling the risk of infection?
  - How did the care home define ‘end of life’ in this context?
    - PROMPT: Such as the last year of life, months or weeks and days?

**Can you think back to when caring for a resident who was deteriorating and ‘sick enough to die’**

- How did you communicate with families when residents were close to dying?
  - What were the challenges?
  - What helped you to address them?

- How were you able to support families who were bereaved?

**7) Advice and support from external services**

I would like to explore what kind of advice and support your care home received from external services in your area, and how that affected your provision of palliative and end-of-life care

- What services outside of the care home supported you to meet residents' health needs?
  - PROMPT: And when a resident was deteriorating? And at the end of life?
  - PROBE: What services and support did you most value? What would you have liked more of?
  - PROMPT: GPs? Community nurses? Community Pharmacy? Paramedics?
- What supported you to work with services outside the care home?
  - PROMPT: GPs, local hospice or palliative care team, community services, like community nurses, hospital services like a geriatrician
- How did you manage to provide palliative and end-of-life care out of hours, such as at the weekend and at night?
  - PROBE: What services supported you? What would you have liked more of?
- What were the innovations in using technology to communicate with services, like the GP to provide care and guide decisions?
    - How effective is remote palliative care? Do you think it works?
    - What resources were needed for effective telehealth?

**8) Impact of COVID-19**

- What has been the impact of COVID-19 on staff?
  - PROMPT: staff turnover, absenteeism, stress, agency staff
- How did this affect the delivery of palliative and end-of-life care?
- What is the best way to provide bereavement support for staff?
- How has COVID-19 impacted your wellbeing?
  - PROBE: What support do you wish you had had for your wellbeing and mental health during this difficult time?

**Closing questions**

- What guidance or training do you wish had been given that would have helped you provide palliative and end-of-life care?
- What do you think should change in order to provide better palliative and end-of-life care in the future?

- Is there anything else you would like to say about your experiences of providing palliative and end-of-life care during the pandemic?
- Is there anything we have talked about that has worried or distressed you that you would like to talk through?
- If something comes to mind you can contact me at …, or please talk with your GP
